# Adolescent Interventions to Manage Self-Regulation in Type 1 Diabetes (AIMS-T1D): randomized control trial study protocol

**DOI:** 10.1186/s12887-020-2012-7

**Published:** 2020-03-07

**Authors:** Alison L. Miller, Sharon L. Lo, Dana Albright, Joyce M. Lee, Christine M. Hunter, Katherine W. Bauer, Rosalind King, Katy M. Clark, Kiren Chaudhry, Niko Kaciroti, Benjamin Katz, Emily M. Fredericks

**Affiliations:** 1 Department of Health Behavior and Health Education, University of Michigan School of Public Health, 1415 Washington Heights, SPH I Room 3718, Ann Arbor, MI 48109-2029 USA; 2 Center for Human Growth and Development, University of Michigan, Ann Arbor, MI USA; 3 Department of Pediatrics, University of Michigan Medical School, Ann Arbor, MI USA; 4 Susan B. Meister Child Health Evaluation and Research Center (CHEAR), University of Michigan, Ann Arbor, MI USA; 5 Department of Nutritional Sciences, University of Michigan School of Public Health, Ann Arbor, MI USA; 6 Office of Behavioral and Social Sciences Research, National Institutes of Health, Bethesda, MD USA; 7 Eunice Kennedy Shriver National Institute of Child Health and Human Development, National Institutes of Health, Bethesda, MD USA; 8 Department of Human Development and Family Science, Virginia Tech, Blacksburg, VA USA

**Keywords:** Self-regulation, Behavioral intervention, Medical regimen adherence, Type 1 diabetes, Adolescence

## Abstract

**Background:**

Self-regulation (SR), or the capacity to control one’s thoughts, emotions, and behaviors in order to achieve a desired goal, shapes health outcomes through many pathways, including supporting adherence to medical treatment regimens. Type 1 Diabetes (T1D) is one specific condition that requires SR to ensure adherence to daily treatment regimens that can be arduous and effortful (e.g., monitoring blood glucose). Adolescents, in particular, have poor adherence to T1D treatment regimens, yet it is essential that they assume increased responsibility for managing their T1D as they approach young adulthood. Adolescence is also a time of rapid changes in SR capacity and thus a compelling period for intervention. Promoting SR among adolescents with T1D may thus be a novel method to improve treatment regimen adherence. The current study tests a behavioral intervention to enhance SR among adolescents with T1D. SR and T1D medical regimen adherence will be examined as primary and secondary outcomes, respectively.

**Methods:**

We will use a randomized control trial design to test the impact of a behavioral intervention on three SR targets: Executive Functioning (EF), Emotion Regulation (ER), and Future Orientation (FO); and T1D medical regimen adherence. Adolescents with T1D (*n* = 94) will be recruited from pediatric endocrinology clinics and randomly assigned to treatment or control group. The behavioral intervention consists of working memory training (to enhance EF), biofeedback and relaxation training (to enhance ER), and episodic future thinking training (to enhance FO) across an 8-week period. SR and treatment regimen adherence will be assessed at pre- and post-test using multiple methods (behavioral tasks, diabetes device downloads, self- and parent-report). We will use an intent-to-treat framework using generalized linear mixed models to test our hypotheses that: 1) the treatment group will demonstrate greater improvements in SR than the control group, and 2) the treatment group will demonstrate better treatment regimen adherence outcomes than the control group.

**Discussion:**

If successful, SR-focused behavioral interventions could improve health outcomes among adolescents with T1D and have transdiagnostic implications across multiple chronic conditions requiring treatment regimen adherence.

**Trial registration:**

ClinicalTrials.gov: NCT03688919; registered September 28, 2018.

## Background

Decades of research have demonstrated the importance of self-regulation (SR), or the capacity to control one’s thoughts, emotions, and behaviors in order to achieve a desired goal, in shaping individuals’ health outcomes [1, 2]. SR capacity has been conceptualized as including three component processes: executive functioning skills; emotion regulation strategies; and ability to delay gratification in service of achieving future goals [3, 4]. Low SR capacity has been shown to interfere with individuals’ engagement in a variety of health maintenance behaviors including engaging in regular physical activity [5], consuming a healthy diet [6–8], and adhering to medical treatment regimens [9–11].

Type 1 Diabetes (T1D) is a condition affecting millions worldwide. For example, in the U.S. about 17,900 children and adolescents younger than age 20 were newly diagnosed with type 1 diabetes during 2011–2012 [12]. Adhering to a recommended treatment regimen is critical in managing glycemic control and establishing long-term health among pediatric T1D patients [13–15]. Yet, poor treatment regimen adherence is common among adolescents [16–18]. Only 21% of adolescents meet the American Diabetes Association guidelines for Hemoglobin A1c (HbA1c) target level of 7.5% [14, 19], and treatment adherence and glycemic control decline across this developmental period [20]. Poor glycemic control places youth with T1D at substantially increased risk of acute health events including hyper and hypoglycemia and diabetic ketoacidosis, as well as serious, long-term comorbidities such as retinopathy, neuropathy, kidney disease, and cardiovascular disease [21].

To achieve and maintain optimal glycemic control, adolescents must adhere to a complex self-care regimen that includes monitoring blood glucose, administering insulin via daily injections or a pump, regulating carbohydrate intake, engaging in regular physical activity, and minimizing both hyper- and hypoglycemia [22, 23]. Each of these are daily tasks that require SR. For example, executive function (EF) skills such as working memory and planning are needed to recall and ensure adherence to dietary and exercise plans [24–26]. Emotion regulation (ER) skills are important for coping with diabetes-related distress, combating depression, and managing feelings of anxiety that interfere with monitoring blood sugar levels [27–29]. Future orientation (FO), or the ability to delay gratification and focus on future goals, may be particularly important for adolescents with T1D in order to adhere to their treatment regimen in the face of competing demands.

Positive associations between SR capacity and treatment adherence are consistently reported in prior work among youth with T1D [3, 30, 31] and poor SR has been suggested as a central mechanism contributing to nonadherence [23–26, 32]. For example, in 13–17 year-olds with T1D, parent-rated EF deficits in working memory and attention were associated with poorer treatment adherence [33], with further research identifying that associations between EF and glycemic control were mediated through treatment adherence [26]. With regard to ER, findings suggest that adolescents with poorer ER skills have higher HbA1c levels, suggestive of poorer glycemic control [34, 35]. Finally, no studies have specifically examined associations between FO and treatment adherence in adolescents with T1D. However, in adults with T1D, results suggest FO skills are positively associated with treatment adherence [36, 37]. Moreover, FO is associated with engagement in several other health behaviors that promote long-term goals [38, 39], including in adolescents [40]. Given this body of research, the pervasiveness of poor treatment adherence among adolescents with T1D, and the fact that the fact that treatment regimens shift from parent-managed to adolescent managed across this period [16, 41–43], improving adolescents’ SR capacity may be an important strategy for helping them better manage treatment adherence tasks [3, 44].

Promoting SR may increase adolescents’ ability to engage in diabetes-specific adherence behaviors and thereby improve T1D outcomes [23, 44–46]. Specifically, improving EF skills could aid adolescents’ remembering regimen details and timing [33], enhancing ER capacity could improve metabolic control by promoting better regulation of stress hormones, reducing diabetes-related distress, and increasing capacity to focus on regimen adherence [11, 47]; and improving FO capacity may increase adherence by engaging adolescents to invest energy in behaviors that lead to long-term health [48, 49]. Yet, improving SR has not been tested as a mechanism of behavior change in this population.

### Study aims and hypotheses

The primary aim of the Adolescent Interventions to Manage Self-Regulation in Type 1 Diabetes (AIMS-T1D) study is to determine whether a behavioral intervention improves SR targets (EF, ER, FO) among adolescents (ages 13–17) with T1D. The secondary aim to determine whether the behavioral intervention improves T1D treatment regimen adherence among participating adolescents. We hypothesize that adolescents randomized to the treatment group will demonstrate significant improvements in EF, ER, and FO compared to adolescents randomized to the control group after 8 weeks of intervention. We hypothesize that adolescents randomized to the treatment group will demonstrate significant improvements in treatment regimen adherence including improved blood glucose monitoring and insulin administration adherence, as well as self-reported treatment regimen adherence).

## Methods/design

### Approval and trial registration

The AIMS-T1D study is a 24-month randomized controlled trial taking place in Ann Arbor, Michigan. At the time of submission of this manuscript, participants were being enrolled into the trial. The trial has been approved by the University of Michigan Institutional Review Board (HUM00148853) and is registered with ClinicalTrials.gov, ID NCT03688919. An internal Data Safety Monitoring Board consisting of pediatric psychologists, pediatric endocrinologists, and adolescent medicine specialists in T1D was formed to monitor the study (e.g., determine stopping rules, review any adverse events).

A total sample of 94 participants aged 13–17 years with T1D will be recruited from a pediatrics diabetes clinic research registry of patients receiving diabetes care at the University of Michigan.

### Inclusion and exclusion criteria

Adolescents must meet the following criteria to participate: 1) been diagnosed with T1D for at least 6 months; 2) aged between 13 and 17 years; 3) reside with a parent/legal guardian who is the primary caregiver; 4) have HbA1c ≥ 7.0; 5) have regular access to Wi-Fi; 6) feel comfortable speaking English enough to complete study activities; and 7) receive diabetes care from providers within Pediatric Endocrinology at Michigan Medicine. All diabetes treatment regimens are included in the study (e.g., multiple dose injection, blood glucose monitoring [BGM], continuous glucose monitoring [CGM], or a combination). Exclusion criteria are: 1) non-fluency in English in parent or child; or 2) psychiatric or cognitive conditions, such as clinically significant depression assessed via phone screen at intake that would impede ability to participate.

### Participant recruitment

Participants will be recruited via email, phone, and text message sent to themselves or their parent(s) using contact information obtained by the Pediatrics Endocrinology clinic. The study will also be advertised in newsletters sent by the clinic and via flyers available in clinic. Eligible participants may also be recruited face-to-face during clinic visits. Participants who communicate interest in study participation will complete a phone screening and eligible participants will have their baseline study visit scheduled. Adolescents will be randomized to intervention group when scheduled. A research assistant will obtain informed consent from parents for their own and their child’s participation in the study; adolescents also provide assent for their participation in the study.

### Randomization strategy and blinding

Participants will be randomized to receive the AIMS-T1D intervention or the control activities (see below) using an Excel-based randomization tool (2 conditions: treatment and control) and a random number generator. To blind evaluation study team members to participants’ condition assignment, the intervention team will be exclusively responsible for creating and implementing the randomization assignments as well as participant case management during the 8-week intervention period.

### Intervention conditions

**Fig. 1:**
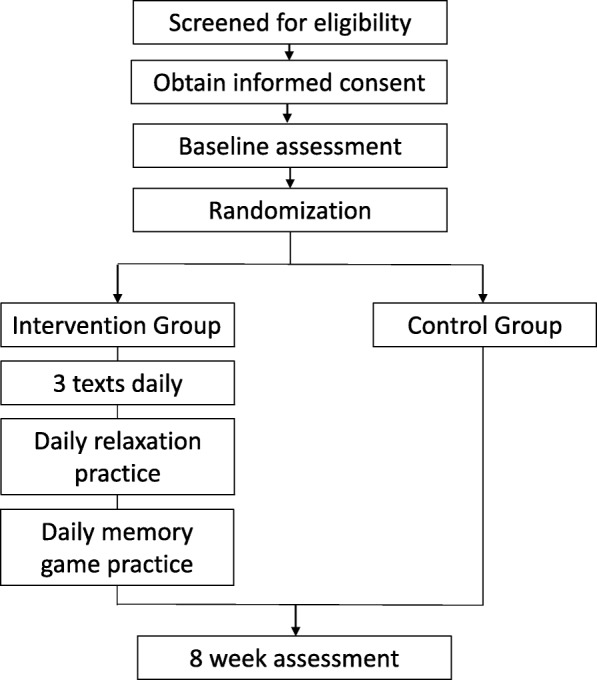
Study procedure details

As recommended by the Standard Protocol Items: Recommendations for Interventional Trials (SPIRIT) Guidelines [50], study procedure details are outlined in Fig. 1 (see Additional file 1 for SPIRIT Checklist). Treatment and control group activities are described below.

### Treatment group activities

**Fig. 2:**
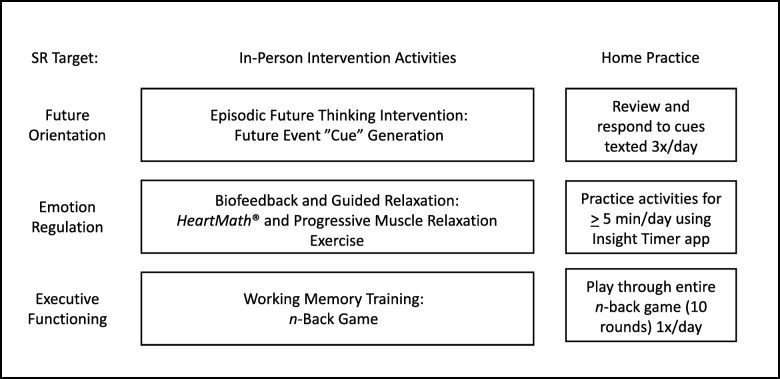
Self-regulation targets and intervention activities

The AIMS-T1D intervention is based on prior work that used a multiphase optimization strategy design [51] framework to test different intervention strategies on SR targets among youth, specifically EF, ER, and FO [4]. Intervention activities, which are manualized, will be introduced to participants in person after randomization by trained interventionists. Sessions will be coded for fidelity from video. Participants assigned to the treatment condition will participate in 8-weeks of home practice; participants will have regular contact with interventionists via text-based reminders to help ensure adherence and practice records will be used to assess intervention dose. A detailed description of activities is below and shown in Fig. 2. Note, as the intervention will be conducted in the order described below, we present intervention activities and measures first for FO, then ER, and then EF.

#### Future orientation

Episodic future thinking (EFT), or projecting to “make the future become the present” can promote FO [38, 39]. We will employ EFT techniques that have been previously used with adolescents [52]. The goal of these activities is to enhance the participants’ ability to “pre-experience” the future in an active, vivid, and concrete manner. A positive personal goal or event is selected, and the feelings and experience of achieving the goal or the event are verbalized and practiced [53]. The interventionist will coach the participant to describe an upcoming, positive event at least 2 months away (i.e., the duration of the study), using present tense and concrete details (e.g., who will be there, where event will take place, visual/sensory information) in order to make the experience as vivid as possible. The participant will generate three such future events, also known as cues. The participant will then be asked to link each cue to their T1D treatment regimen (i.e., “because I checked my blood glucose level on my way to my friend’s house and was able to give insulin to bring my blood glucose in range, I felt more confident spending the evening there”). For home practice, text messages with these cues will be sent at 3 preferred times of day; participants will review the cue and answer on a scale of 1–5 how vividly they are able to bring it to mind.

#### Emotion regulation

Evidence-based biofeedback and guided relaxation techniques will be used to promote ER [54, 55]. The interventionist will use a computer-based program, *HeartMath*® to show the participant how to use diaphragmatic breathing to improve heart rate variability coherence [56]. The interventionist will guide the participant to match the pace of their breath to a graphic of a Mandala expanding and contracting (5 min) and how to use diaphragmatic breathing to change a picture of a garden from black-and-white to multi-colored (greater coherence, more of the garden becomes colored). The final activity is a progressive muscle relaxation exercise. ER home practice consists of 5 guided meditations that incorporate diaphragmatic breathing (using Insight Timer, a free meditation app downloaded on the participant’s phone), to be practiced once a day for 5 min.

#### Executive function

A tablet-based working memory game based on the *n*-back task will be used to improve EF [57, 58]. This task has shown to improve working memory and also to facilitate transfer effects to other aspects of EF, including inhibitory control [59]. This object-based variant of the *n*-back displays sequential images (e.g. fruit, animals) and prompts the participant to tap the screen when an image matches a previous. The game becomes more challenging as the participant completes items accurately, requiring matches of two, three, four, etc. images “back”. The game will be introduced by the interventionist and the participant instructed to practice daily on the tablet (10 rounds of the game, approximately 15 min in total per session), sending screenshots to the interventionist in order to earn incentives (below).

#### Incentives

Participants will be incentivized to complete home practice activities with the opportunity to earn $10 a week for reviewing and completing the one-item cue question described above for at least 12 of the 21 cues they received via text message, and texting four screenshots of meditation practice and four screenshots of the *n*-back game to the interventionist. Additionally, each screenshot translates to 1 raffle ticket for an Amazon gift card, with an opportunity to earn 2 bonus raffle tickets if every home practice task is completed.

### Control group activities

Control group activities consist of enhanced usual care. After randomization, participants assigned to the control condition will meet with an interventionist to receive an interactive PowerPoint presentation reviewing resources from the University of Michigan Pediatric Diabetes clinic website (www.umpedsdiabetes.com), to which all clinic families have access. For the 8-week study period, in lieu of AIMS-T1D intervention activities, participants will receive 1 text message per week with a link to the clinic website and be asked to explore the pages of interest to them. The text messages will be sent at the same time and day each week, selected by the participant.

### Study measures and assessment timeline

Measures of all primary and secondary outcomes will be conducted at baseline and again 8 weeks later in order to evaluate change in each outcome among participants in the intervention versus control groups (see Table 1 for list of measures and assessment method). Staff are extensively trained in both questionnaire and behavioral task administration using manuals developed in prior work that used similar assessment strategies [4].

**Table 1:** Primary and Secondary outcome measures and assessment method

	Assessment method
**Primary outcomes**	
Future orientation	
Delay discounting 5-trial task	Behavioral task
Consideration of future consequences scale	Self-report
NIH toolbox self-efficacy scale	Parent and self-report
Emotion regulation	
Structured interview for disorders of extreme stress affect dysregulation scale	Self-report
Positive and negative affect schedule	Self-report
NIH toolbox perceived stress survey	Parent and self-report
Executive function	
Forward/Backward digit span task	Behavioral task
Go-No/Go task	Behavioral task
Behavior rating inventory of executive functioning, 2nd Ed.	Parent and self-report
**Secondary outcomes**	
Blood glucose monitoring	Diabetes device
Insulin administration adherence	Diabetes device
Self-care inventory revised	Parent and self-report

### Primary outcome measures (SR)

Primary study outcomes include FO, ER, and EF as indicators of SR. Where appropriate, SR is assessed using a combination of direct assessment (behavioral tasks completed by the participant) plus adolescent self-report and parent-report on standard questionnaires that have been found to be reliable in prior work [4]. Task and questionnaire-based assessments will be analyzed separately as such measurement approaches may represent different aspects of SR. [60]

#### Future Orientation (FO)

The degree to which one discounts the future is an aspect of FO that will be measured using the 5-trial Delay Discounting Task [61]. Each trial uses one monetary amount (e.g., $1000 to $1000,000). Each participant is asked on the first trial whether they would prefer to receive that amount in 3 weeks or half that amount now. On the next trial the question is repeated but with a different time delay according to the participant’s response on the previous trial. That is, a greater delay is presented on the next trial if the participant chose “now” on the previous trial, whereas a lesser delay is presented if the participant chose the later time on the previous trial. The dependent measure is the steepness of the delay discounting curve; steeper curves indicate that an individual is less future-oriented (i.e. more impulsive).

Considering the future and how one’s actions can affect future consequences is an aspect of FO that will be measured by youth self-report using the Consideration of Future Consequences Scale [62]. Adolescents will answer 14 questions (e.g., “I think about how things would be in days to come, and try to influence those things in my daily behavior”) on a 7-point scale (1 = Not at all like me to 7 = Very much like me). Higher scores indicate a greater consideration of future consequences or future-oriented behavior. Self-efficacy is hypothesized to promote future-oriented thinking, and is thus an aspect of FO that will be measured using a composite of the National Institutes of Health (NIH) Toolbox Self-Efficacy parent report and the self-report form (10 items each) [63]. Participants respond to questions about their child’s or their own (in the case of the child) self-efficacy. Mean scores are generated; higher scores are indicative of greater perceived self-efficacy.

#### Emotion Regulation (ER)

The adolescent’s ER will be measured using self-reports of dysregulated affect using a 6-item scale based on the Structured Interview for Disorders of Extreme Stress [64] (items are averaged to indicate greater affect dysregulation, range: 1–6); and self-reports of emotion experiences on the 20-item Positive and Negative Affect Schedule [65] (items are summed to indicate more negative [10 items] and fewer positive experiences [10 items]; range: 10–50). A composite measure of parent- and self-reports on the Perceived Stress Survey [63], a 10-item measure of stress in children and youth (items are summed to indicate greater perceived stress; range: 0–40) will also be used. A composite measure will be created by standardizing and averaging affect dysregulation, emotion experience, and perceived stress scores. It will be scored such that higher values indicate poorer ER.

#### Executive Function (EF)

EF will be measured using standard tasks (Forward/Backward Digit Span, Go-No/Go). Digit span assesses working memory, a key component of EF. In Digit Span [66], participants repeat numbers that the examiner reads aloud in order or reverse order (8 questions, 2 trials each; correct response is 1 point; incorrect or no response is 0 points). Scores are summed for each trial; maximum total raw score is 16 points. The Go-No/Go task [67] is presented on a laptop and assesses inhibitory control, another key component of EF. Participants hit a key to respond when they see the ‘go’ stimulus (presented for 300 ms) but not when they see the no-go stimulus. Go-No/Go responses are scored based on reaction time (seconds) and accuracy (0–100%). A composite variable indexing better behavioral EF based on Digit Span and Go-No/Go tasks will be created by generating standardized z-scores for each task variable and calculating a mean score.

EF will also be assessed using the parent- and self-report versions of the Behavior Rating Inventory of Executive Functioning, 2nd Edition, a standardized EF measure [68]. Subscales assess ability to control impulses, flexibly change direction, pay attention, modulate responses, and anticipate events. Items are combined to form a Global Executive Composite score, which is a standardized score representing overall EF difficulties (range 0–100). Self- and parent reports will be used to measure overall Global Executive Functioning, scored such that higher scores indicate greater difficulties in EF functioning.

### Secondary outcome measures (treatment regimen adherence)

Secondary outcomes assess adherence to the participant’s T1D regimen, specifically blood glucose monitoring (BGM), insulin administration, and scores on the Self-Care Inventory Revised (SCI-R) [69]. The continuum of adolescents’ adherence will be examined, as well as changes in the proportion of adolescents “adherent” vs. “nonadherent” based on clinical criteria [70].

BGM frequency will be assessed by downloading data from the prior 2 weeks from the adolescent’s glucometer or relevant blood glucose monitoring device (e.g., CGM). Adherence to BGM is defined as an average of 4 blood glucose measurements/day and/or wearing a CGM pump 6 out of 7 days. Insulin administration adherence is defined as at least 3 short acting insulin boluses/day. Insulin administration frequency for participants an insulin pump can be measured using data from software downloads (e.g., Glooko/diasend, Tidepool). For participants on injections, the number of short-acting insulin boluses will be measured by self-report.

Finally, the SCI-R [69] is a 14-item self- and parent-report measure of multiple T1D self-care adherence behaviors. Items reflect main aspects of the T1D regimen, including: monitoring and recording glucose, administering and adjusting insulin, regulating meals and exercise, and keeping appointments. Respondents report on adherence behaviors on a 5-point scale (1=“never do it”; 5=“always do this as recommended without fail”; or N/A). Adolescent and parent responses will be summed and analyzed separately.

### Data analysis

After secure data entry, coding, cleaning, and creating and checking the psychometric properties of any composite variables, we will conduct our study analyses. We will use an intent-to-treat analytic framework to test whether the AIMS-T1D intervention was effective in changing SR targets (primary outcome) and treatment regimen adherence (secondary outcome) variables. We will compare change in the group assigned to treatment compared to the group assigned to the control condition from pre- to post-test across the 8-week intervention period. All analyses will use an alpha value of *p* < .05 and will be conducted in SAS. Baseline comparability of the groups will first be assessed using bivariate analyses (t-tests, X^2^) as appropriate.

We will conduct our primary and secondary outcome analyses using linear mixed effects models (for continuous outcomes) and generalized linear mixed models (for dichotomous outcomes) to compare changes in SR and adherence across the two groups (treatment vs. control). The mixed effects models will account for the within-subject correlations due to having repeating measurements in individual subjects over time. Both unadjusted and adjusted analyses will be performed, with adjustment for important baseline covariates that relate to the outcome, including participant age and sex.

We anticipate that some participants will have only partial adherence to the AIMS-T1D intervention (i.e. complete only some home practice sessions), thus we will also conduct a dose-response analysis where dose corresponds to number of sessions, controlling for covariates that relate to dosage and outcome of interest.

#### Power

We based our power analysis on prior work detecting change in SR targets [4] and T1D medication adherence literature [10, 11]. We anticipate medium effect sizes of 0.6 with the bundled interventions, so our proposed sample size of 94 (47 per group) should result in sufficient statistical Power of 82% to detect such effects using a two-sided Type I error alpha of 0.05. In addition, controlling for baseline variables that relate to outcome will reduce the residual variance and further increase statistical Power.

## Discussion

Given that only about a fifth of adolescents with T1D meet recommended T1D treatment targets, there remains an urgent need to identify effective strategies to improve treatment regimen adherence among this population. As responsibility for diabetes management shifts from parent to child across the adolescent period, identifying interventions that are engaging and effective for this developmental stage is essential. Difficulties with SR may underlie poor T1D treatment regimen adherence during adolescence, a time characterized by continued SR maturation and daily challenges to SR due to typically developing demands (e.g., social, academic, emotional). Indeed, developmental neuroscience suggests adolescence is a time of uneven growth in SR skills such as EF, but is also characterized by increasing ability to take broader perspectives and envision the future, including health behaviors; thus, adolescence is a unique and compelling period for intervention [71]. Every day, adolescents with T1D must engage in tasks that require a high degree of SR, including monitoring blood sugar and carbohydrate intake, maintaining a schedule for eating as well as physical exercise, and ensuring adequate access to T1D supplies and resources in the event that blood sugars are too high or too low. Further, as psychological adjustment to and coping with T1D can also interfere with treatment adherence [27], ER strategies may play a critical role in promoting adherence. Finally, adhering to T1D regimens requires FO, which allows adolescents to understand and appreciate how achieving current adherence goals, which may be difficult, can help maintain optimal HbA1c levels in order to avoid long-term complications.

The current randomized control trial proposes to test whether the AIMS-T1D behavioral intervention can improve adolescents’ SR and treatment regimen adherence. The study takes an experimental medicine approach to behavior change by testing the impact of an intervention targeting SR as a primary outcome [72]. As prior work using similar interventions suggests that these aspects of SR are malleable [52, 54, 59], the next step in a systematic experimental medicine approach to building and testing better interventions is to test whether these early findings can generalize to a new population, specifically adolescents with T1D. A benefit of this approach is that by identifying whether the interventions designed to change SR actually do so, we will gain critical information about the utility of such interventions for future research in new populations. To our knowledge, this is one of the first studies to test a SR-focused intervention in youth in relation to a health-relevant outcome. From a prevention perspective, it is more cost-effective to prevent negative health outcomes associated with poor treatment regimen adherence than to treat the long-term sequelae of poor adherence. Further, by improving SR during adolescence, we may achieve not only better T1D treatment regimen adherence in the short term, but impact lasting improvements both in SR and the health trajectory of those with T1D. From a trans-diagnostic perspective, if this trial demonstrates meaningful improvements in adolescents’ SR, the intervention may be a novel approach to health behavior change among other populations of adolescents with medical needs that require strict adherence to treatment regimens. Therefore, findings from the AIMS-T1D study not only have the possibility of informing our understanding of SR as a mechanism of behavior change among adolescents with T1D, but also have implications, and thus the potential, for broad impact.

## Supplementary information

**Additional file 1.** SPIRIT Checklist: Recommended items to address in a clinical trial protocol and related documents.

## Data Availability

The datasets used and/or analyzed during the current study are available from the corresponding author on reasonable request.

## Abbreviations

AIMS-T1D: Adolescent Interventions to Manage Self-Regulation in Type 1 Diabetes
BGM: Blood Glucose Monitoring
CGM: Continuous Glucose Monitoring
EF: Executive Function
EFT: Episodic Future Thinking
ER: Emotion Regulation
FO: Future Orientation/Motivation
HbA1c: Hemoglobin A1c
NIH: National Institutes of Health
SCI-R: Self-Care Inventory Revised
SR: Self-regulation
T1D: Type 1 Diabetes
